# Filter, Flip, and Fabricate: A Wax-Assisted Stamp-Transfer Approach for Flexible Ti_3_C_2_T_*x*_ MXene Electrochemical Transducers

**DOI:** 10.1021/acsami.5c24165

**Published:** 2026-01-15

**Authors:** Zaheer Ud Din Babar, Andy Bruno, Gabriel Maroli, Syedah Afsheen Zahra, Bartolomeo Della Ventura, Raffaele Velotta, Vincenzo Iannotti, Ruslán Alvarez-Diduk, Arben Merkoçi

**Affiliations:** † 231882 Catalan Institute of Nanoscience and Nanotechnology (ICN2), CSIC and BIST, Campus UAB, Bellaterra, Barcelona 08193, Spain; ‡ Scuola Superiore Meridionale (SSM), University of Naples Federico II, Largo S. Marcellino, 10, Naples 80138, Italy; § Department of Physics “E. Pancini”, 154931 University of Naples Federico II, via Cintia 26, Naples 80126, Italy; ⊥ Autonomous University of Barcelona (UAB), Bellaterra, Barcelona 08193, Spain; ∥ Laboratory for Functional Polymers, EMPA Swiss Federal Laboratories for Materials Science and Technology, Überlandstrasse 129, Dübendorf 8600, Switzerland; ∇ CNR-SPIN c/o Department of Physics, 9307 University of Naples Federico II, Piazzale V. Tecchio 80, Naples 80125, Italy; ○ ICREA Catalan Institution for Research and Advanced Studies (ICREA), Barcelona 08010, Spain

**Keywords:** MXenes, transducer fabrication, device design, electrochemical sensors

## Abstract

Combining additive-free solution processing of MXenes with template-assisted transfer represents an innovative approach for the fabrication of electrochemical sensors. Herein, we introduce a wax-assisted templating and stamp-transfer (*WAST*) approach to fabricate monolithic MXene transducers. To this end, two configurations were developed: (A) *MXene-at-all electrodes*, where the working, counter, and reference electrodes were entirely composed of MXene, and (B) MXene working electrodes combined with an Ag/AgCl pseudoreference and carbon counter electrodes. Additive-free MXene inks, at different concentrations and volumes, were filtered onto wax-templated PVDF membranes to form stampable patterns. Voltammetric characterization revealed consistent anodic and cathodic peak currents (*I*_pa_ and *I*_pc_) and steady peak-to-peak separation *(*Δ*E*_p_) across three independent batches, indicating excellent reproducibility with *I*_pa_ = 87.9 ± 1.7 μA, *I*_pc_ = −81.3 ± 1.5 μA, and Δ*E*_p_ = 255.9 ± 4.6 mV for configuration A, and *I*_pa_ = 43.8 ± 0.6 μA, *I*_pc_ *=* −62.8 ± 1.2 μA, with a 3-fold lower peak separation (Δ*E*_p_ = 87.6 ± 0.9 mV) in the case of configuration B (mean ± SE, *n* = 15). Primary benchmarking with similar commercial systems signifies the efficacy of *WAST*-produced transducers to mimic commercial electrode performance and provides a robust alternative to conventional deposition methods. In addition, a proof-of-concept experiment for Hg^2+^ ion sensing validated its potential for practical applications. The *WAST* method offers a robust fabrication, configuration choice, and tool-light patterning that can accelerate lab-scale prototyping of stand-alone flexible electrochemical devices.

## Graphical abstract

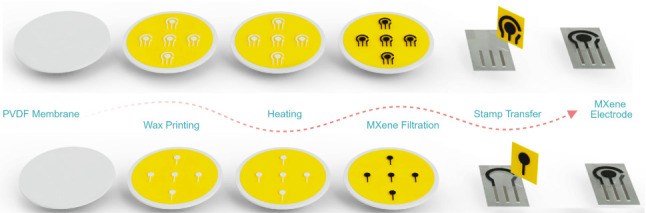

## Introduction

The rise of the *“do-it-yourself”* culture has profoundly accelerated the prototyping of miniature electronic devices and their integration into sensor research. This democratization of eco-design development, in compliance with the United Nations Sustainable Development Goals (SDGs),^1,2^ has simultaneously intensified the demand for material- and energy-efficient fabrication approaches. Among conventional patterning approaches, inkjet printing is low-cost and material-efficient,^3^ yet it frequently relies on formulated inks and postprocessing, with chemical/thermal steps often needed to obtain conductive, nanostructured films.^4,5^ When surfactant- and polymer-free aqueous processing is required, vacuum filtration offers a complementary route that maintains benign solvents and affords precise control of mass loading and pattern fidelity.^6–8^ Coupling it with WAX-assisted templating has enabled customizable patterning of two-dimensional (2D) nanomaterials with micro- to nanometer-scale precision.^9–11^ Transition metal carbides (known as MXenes),^12^ a distinct class of 2D materials extracted from MAX phases (where M is an early transition metal, A is an element from groups 13 or 14, X represents carbides, nitrides, or both),^13^ conventionally rely on existing current collectors, e.g., glassy carbon electrode (GCE) to investigate their sensing capability. With a few exceptions,^14,15^ their integration into printed configurations typically involves drop-casting,^16,17^ which requires binders and can lead to uncontrolled morphology and masking of intrinsic electroanalytical behavior. Also, the inability of drop-casting to achieve well-defined patterns or coatings leads to reduced device-to-device reproducibility. Other established methods entail a critical trade-off: spray or spin coating of low-concentration MXene dispersions results in substantial material waste,^18^ whereas the use of polymeric additives as rheological modifiers can inevitably compromise their essential features.^19,20^ This underscores the pressing need for an eco-design strategy with near-zero waste and additive-free deposition of aqueous MXene dispersions on a single substrate to effectively streamline the prototyping of MXene-based sensors.

Here, we report an approach for the direct fabrication of monolithic, additive-free MXene electrodes through a wax-assisted stamp-transfer *(WAST)* process. *WAST* is a *“filter, flip, and fabricate”* method that uniquely integrates facile patterning, vacuum filtration, and dry stamp transfer for high-resolution electrode architectures on arbitrary substrates (e.g., PET, paper) with monolayer-to-multilayer thickness control. The process consisted of four steps: (i) sequential screen printing of connectors and counter/reference electrodes, (ii) wax patterning of the target geometries onto the filtration membrane, (iii) vacuum filtration of MXene ink, and (iv) stamp transfer of the patterns onto the substrate. This strategy offers distinct advantages; in particular, its self-regulating nature intrinsically regulates film thickness through precise control of the filtrate’s volume and concentration. By employing *WAST*-fabricated MXene electrodes and systematically evaluating different electrode configurations through voltammetric analysis, we bridged the gap between lab-scale MXene synthesis and local prototyping of EC sensing devices. In addition, this approach not only preserves the innate properties of MXenes but also enables the study of their intrinsic nature as a stand-alone transducer, a capability crucial for the development of next-generation, fully integrated sensing platforms. *WAST* method, which is central to this study, can potentially expand the application landscape of MXenes to flexible and disposable electrochemical transducers.

## Results and Discussion

### MXene Synthesis and Ink Formulation

The topochemical conversion of MAX to MXene is illustrated at the atomic level in Figure 1a, while the detailed protocol for etching and delamination is provided in the Experimental Section. Briefly, Ti_3_AlC_2_ MAX powder was soaked in an etching solution that progressively dissolved the interleaved Al layers and produced multilayered Ti_3_C_2_T_*x*_ *(ml-Ti*_3_ *C*_2_ *T*_*x*_).^21^ Here, *T*_*x*_ denotes surface terminations on the outermost transition metal atoms induced during the etching process.^22,23^ *ml-Ti*_3_ *C*_2_ *T*_*x*_ was subsequently delaminated into single-layer *Ti*_3_ *C*_2_ *T*_*x*_ *(denoted as d-Ti*_3_ *C*_2_ *T*_*x*_) via Li^+^ intercalation. The synthesis of MXene was monitored visually at different stages of its synthesis (Figure 1b) and its quality was initially evaluated through optical analysis, e.g., UV–visible spectroscopy, dynamic light scattering (DLS), and zeta potential (S1). Characteristic absorbance peaks around 270 and 320 nm, with an additional peak at ∼780 nm in the near-infrared region, indicate high-quality conductive material, and negative ζ-potential (−46 mV) confirms its colloidal stability.^24,25^ The observed average hydrodynamic diameter (*d*_Hyd._) of ∼1139 nm and polydispersity index of 0.520 are typically attributed to MAX fragmentation into smaller flakes during synthesis or postprocessing. XRD, SEM, TEM, and XPS further validate the quality of synthesized Ti_3_C_2_T*_x_*. The disappearance of the characteristic Al (104) peak at 2θ ∼39° confirms aluminum removal and successful transformation of Ti_3_AlC_2_ to *ml-Ti*_3_ *C*_2_ *T*_*x*_ and then to *d-Ti*_3_ *C*_2_ *T*_*x*_ (Figure 1c). Whereas the broadening and shifting of the (002) peaks to lower angles (from 2θ ∼ 9.6° to 6°) reflect an increase in the c-lattice spacing from 18.8 to 29.42 Å.^25,26^ The replacement of strong M-A metallic/covalent bonds with weaker van der Waals interactions/hydrogen bonds and terminations on the outermost Ti atoms further increase the interlayer spacing.^27,28^ The delaminated MXenes reassembled into vacuum-filtered films indicated an ordered in-plane crystallographic registry with (00*l*) reflections. Additionally, Li^+^ intercalation corresponds to a reduced interlayer distance (*d*-spacing) of ≈13.1 Å (c-Lp ∼ 26 Å), as the (002) peak shifted to a higher angle (2θ ∼ 6.6°).^26,29^

**Figure 1:**
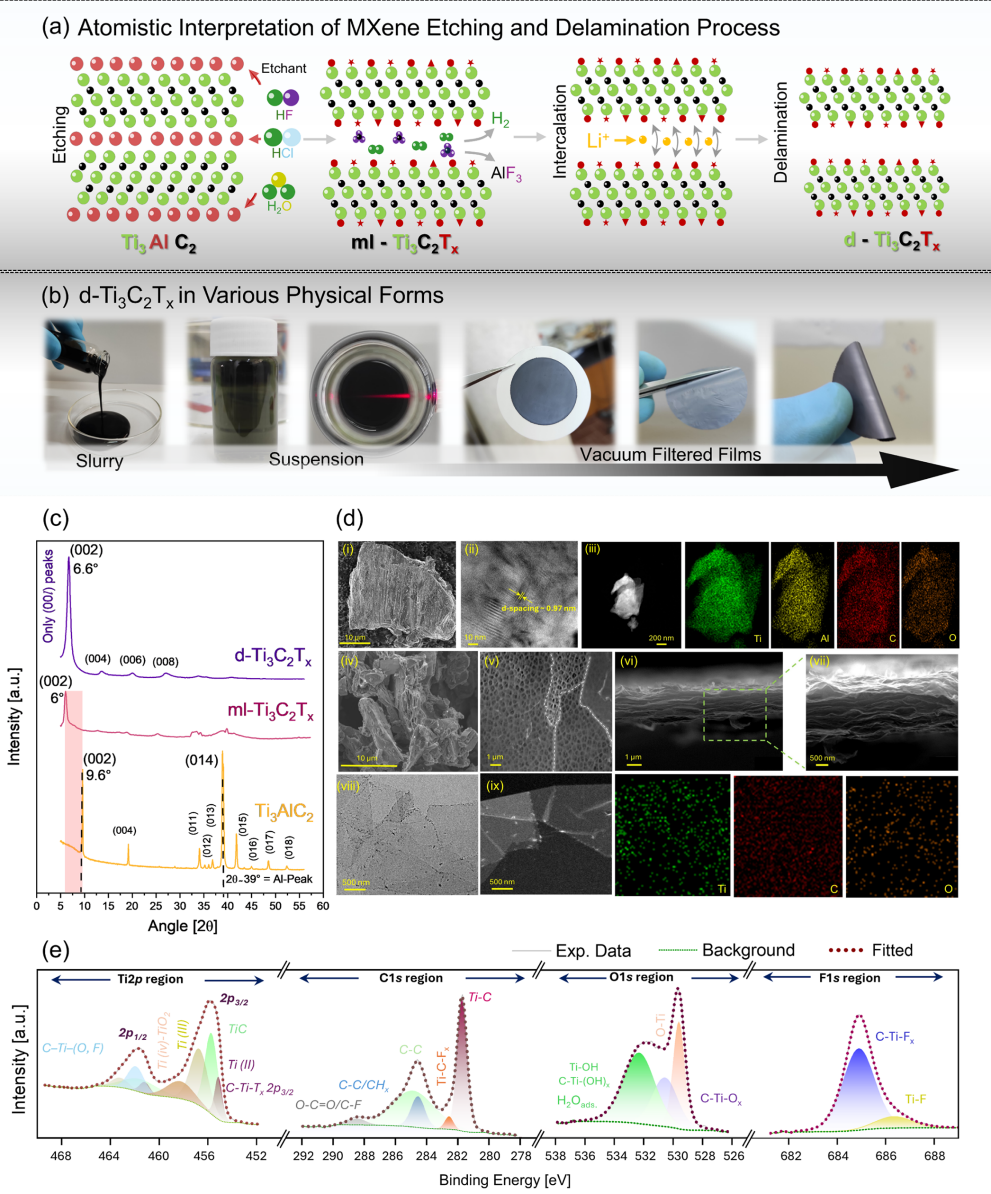
(a) Atomistic illustration of Ti_3_AlC_2_ etching to *ml-Ti*_3_ *C*_2_ *T*_*x*_ and its delamination *(d-Ti*_3_ *C*_2_ *T*_*x*_). (b) Digital photographs of various physical forms of *d-Ti*_3_ *C*_2_ *T*_*x*_ MXene ranging from concentrated to diluted suspensions (with a characteristic green color and Tyndall effect) and free-standing vacuum-filtered films. (c) XRD patterns of *Ti*_3_ *AlC*_2_ MAX, *ml-Ti*_3_ *C*_2_ *T*_*x*_, and the *d-Ti*_3_ *C*_2_ *T*_*x*_ films. (d) Morphological and elemental characterizations: (i) Top-view SEM image, (ii) TEM image, and (iii) Elemental mapping of *Ti*_3_ *AlC*_2_ MAX; (iv) Top-view SEM image of *ml-Ti*_3_ *C*_2_ *T*_*x*_; (v) Top-view SEM of *d-Ti*_3_ *C*_2_ *T*_*x*_ drop-casted on a porous AAO substrate; (vi-vii) Cross-sectional SEM images of the free-standing MXene film; (viii) TEM image; and (ix) STEM image and elemental mapping of d-Ti_3_C_2_T_*x*_. (e) High-resolution XPS spectra with component fitting for the Ti*2p*, C*1s*, O*1s*, and F*1s* regions.

The SEM micrograph (Figure 1d-i) shows a large MAX crystal with a compact, layered structure, while the TEM image shows lattice fringes with a *d*-spacing of ∼0.97 nm (Figure 1d-ii), which is consistent with the XRD results. Furthermore, elemental mapping (Figure 1d-iii) confirmed the uniform distribution of the constituent elements. After topochemical conversion, a multilayered structure with less pronounced accordion-like morphology can be observed (Figure 1d-iv) as the slower kinetics due to low HF concentrations and subsequently lower H_2_ evolution lead to reduced morphological expansion.^26^ In contrast, the top-view SEM images display electron-transparent nanometer-thick delaminated flakes with no observable defects (Figure 1d-v), which was further confirmed by the TEM images (Figure 1d-viii).^30^ Furthermore, the cross-sectional view of the vacuum-filtered films exhibits a well-assembled assembly of isolated flakes (Figure 1dvi–vii).^31^ Notably, the absence of Al content in the STEM elemental maps (Figure 1d-ix) and EDX profiles in S2 proves that any residual aluminum was removed during the delamination.

X-ray photoelectron spectroscopy (XPS) gives further insight into the chemical composition of the flakes. Distinct peaks for C1*s* (∼284.2 eV), Ti2*p* (∼455 eV), O1*s* (533.8 eV), and F1*s* (685 eV), along with Cl and F/O KLL Auger peaks, were observed in the survey spectra (see S3).^32^ High-resolution Ti2*p* spectra split into 2p_3/2_ and 2p_1/2_ components corresponding to Ti–C, Ti­(II), and Ti­(III) at binding energies (BE) near 455, 456.9, and 456.0 eV, respectively (Figure 1e).^33^ Explicitly, C–Ti–T_*x*_ peaks at ∼455.7 eV (2p_3/2_) and ∼461.8 eV (2p_1/2_) indicate the presence of =O and −F terminations, while BE shifts reflect their local bonding.^34,35^ The Ti*2p*_3/2_ peak at ∼458.3 eV is associated with Ti^4+^ and indicates TiO_2_ (or TiO_2–*x*_F_*x*_) formation as well as spontaneous surface oxidation.^36,37^

The C1*s* spectrum contains graphitic C–C (∼284.3 eV), Ti–C (∼281.8 eV), and a minor C–F component at ∼288.1 eV, suggesting high-quality d-Ti_3_C_2_T_*x*_ (Figure 1e).^38,39^ The O1*s* envelope was resolved into lattice TiO_2_ (532.3 eV), C–Ti–O_*x*_ (529.6 eV), C–Ti–(OH)_*x*_, and surface Ti–OH/H_2_O (532.4 eV), indicating abundant surface −OH groups, likely from adsorbed water (Figure 1e).^34,40^ Additionally, the surface Ti–C bonds exhibit significant Ti–O contributions, with the O content associated with adsorbed water molecules.^41^ These observations further support the presence of excessive −OH groups, inducing more negative ζ-potential and supporting the pronounced hydrophilicity (water solubility) of d-Ti_3_C_2_T_*x*_.^42,43^ The F1*s* spectrum displays Ti–F (∼685 eV) and C–Ti–F_*x*_ (∼684.9 eV) peaks^33^ confirming that the surface terminations primarily consisted of =O, −OH, −F, and −Cl groups (Figure 1e). Qualitatively, XPS suggested a higher contribution from physiosorbed −OH groups, with −F and =O acting as chemisorbed terminations.^44^ It should be noted that these functional groups confer highly negative and exceptionally hydrophilic surfaces, consistent with the dispersion stability and electrochemical activity of d-Ti_3_C_2_T_*x*_.

### *WAST* Approach

*WAST* involves the patterning of device architectures as a thin layer of wax onto a filter membrane, followed by the filtration of the active material through it.^45^ Briefly, two configurations were employed: (i) *Configuration A* (named as *MXene-at-all electrodes*), in which silver lines as electrical connectors were screen-printed on a PET substrate, after which MXene layers were transferred. (ii) *Configuration B (Hybrid System)*, where silver connectors, Ag/AgCl pseudoreference electrodes (pRE), and a carbon counter electrode (CE) were sequentially printed (Figure 2a-i). The area designated for the working electrode was intentionally left blank for MXene stamping. Simultaneously, the designs were patterned on a hydrophilic PVDF membrane (pore size = 0.1 μm; diameter = 47 mm) using a Xerox ColorQube 8580 and heated for 10 min at 100 °C (Figure 2a-ii). During heating, the melted wax diffused into the membrane pores and created hydrophobic barriers. Subsequently, vacuum filtration of the additive-free *d-Ti*_3_ *C*_2_ *T*_*x*_ inks at various concentrations (0.5, 0.25, and 0.1 mg·mL^–1^ in water) and volumes (1, 2, and 4 mL) was performed to obtain stampable patterns. Briefly, during filtration, the MXene ink passed only through the unwaxed hydrophilic regions, leading to its collection in a precise shape.

**Figure 2:**
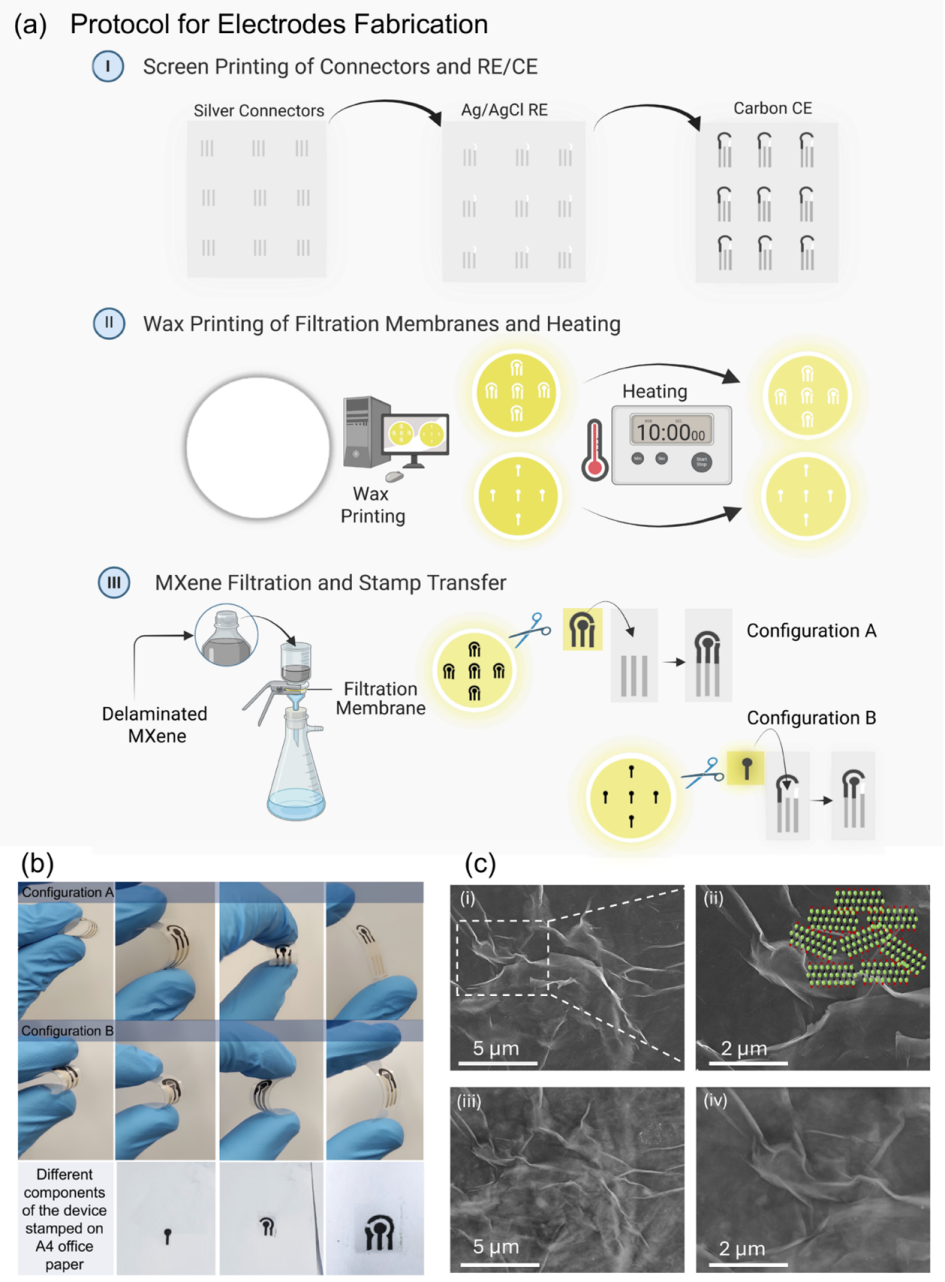
(a) Systematic fabrication of transducers: (i) screen printing of connectors and CE/RE on a PET substrate, (ii) wax layer deposition and heating, and (iii) vacuum filtration of MXene suspensions and subsequent stamp transfer, https://BioRender.com). (b) Digital photographs of stamped electrodes on two distinct substrates. (c) Top-view SEM images of stamped WE with secondary (i–ii) and backscattered electron images (iii–iv) at 5 and 2 μm magnifications, respectively, while the inset depicts projected arrangements of flakes.

After *filtration*, a single electrode was dissected from the membrane, *flipped* onto the oxygen-plasma-activated PET substrate, stamped, insulated with dielectric ink, and dried overnight (Figure 2a-iii). Further details are provided in the Experimental Section. Digital photographs of the stamped electrodes on the PET substrate and standard A4 office paper are presented in Figure 2b. Microscopic analysis revealed a wrinkled and well-adhered interconnected network of uniformly overlapped nanosheets without evident defects (Figure 2ci-ii). Additionally, the surface remained pristine after stamping, as suggested by the backscattered images (Figure 2ciii-iv). Stamped electrodes exhibited low two-point resistance (∼60 Ω) measured between the WE and the silver edge contact (S6), consistent with a tightly packed Ti_3_C_2_T_*x*_ flake network and good interfacial contact.^46^ We note that this 2-wire measurement includes probe/contact contributions and is therefore used as a continuity/quality-control metric. Here, it should also be noted that the amount of active material critically affects the electrochemical performance of a device (herein, we utilized UV–visible spectroscopy to quantify the concentrations used).^25^ To that end, electrodes at various concentrations (0.5, 0.25, and 0.1 mg·mL^–1^) and volumes (1, 2, and 4 mL) were fabricated, and electrochemical performance was evaluated.

### Electrochemical Performance Evaluation

For electrochemical analysis, 5 mM Hexaammineruthenium-III chloride (*RuHex*) with 0.1 M potassium chloride (KCl) was used as the appropriate electrolyte. The electrochemistry of terminal-rich pristine MXenes distinct from that of inert carbon counterparts, in which redox peaks coexist with its pseudocapacitive behavior.^47,48^ Also, the fabrication route and the physical attributes of the device control its ion diffusion pathways and affect electrochemical behavior. Therefore, it requires a thorough optimization of the fabrication process to obtain efficient transducers.

### Configuration A (MXene-at-All Electrodes)

The CV trends in Figure 3a exhibit broad redox peaks and a broadened capacitive envelope that changes with varying MXene concentrations.^49,50^ Similarly, there is an increasing separation between the anodic and cathodic peaks (Δ*E*_p_). As mentioned before, the surface-limited redox reactions and ion diffusion channels in the stacked layers govern the electrochemical behavior of MXenes.^51^ Higher concentrations (e.g., 0.5 mg·mL^–1^) result in densely packed electrodes with plentiful interaction sites for positively charged electrolyte ions to accumulate at the electroactive sites.^47,52^ The electrodes offer abundant charge storage capacity and a substantial capacitive response that overshadows the Faradaic contribution, resulting in its inaccurate quantification.^52,53^ Therefore, electrodes fabricated with higher MXene content lead to redox irreversibility, as supported by elongated voltammograms (with increased Δ*E*_p_ values; Figure 3b).^47,52^ In contrast, reducing the concentration to 0.25 and 0.1 mg·mL^–1^ proportionally refines the Faradaic peaks with less capacitive contributions. Less dense electrodes (or thinner electrodes) produced at lower concentrations expose more flakes to the electrolyte, making the redox process kinetically more active.^54,55^ This underscores the competing influence of concentrations on the modulation of the electrochemical behavior of such systems. Nonetheless, the consistent electrochemical behavior of the individual devices fabricated at 0.5 mg·mL^–1^ from a single batch (Figure 3c) indicates the robustness of the *WAST* method to produce pristine-MXene-based stand-alone transducers.^56^

**Figure 3:**
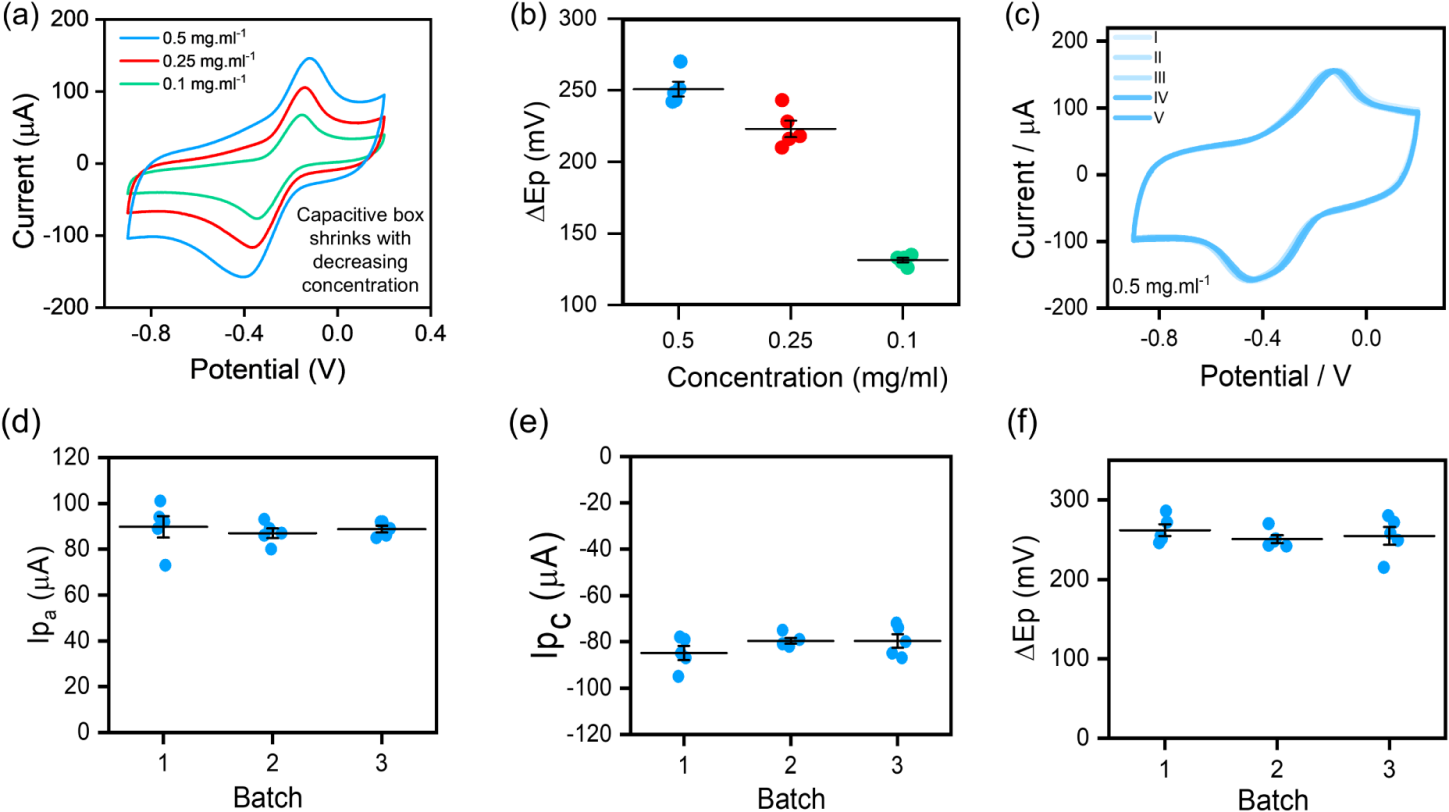
(a) CV trends of electrodes fabricated at different concentrations (0.5, 0.25, and 0.1 mg·mL^–1^); (Scan rate = 50 mV·s^–1^, Electrolyte: 5 mM RuHex in 0.1 M KCl). (b) Δ*E*_p_ variations versus concentration. (c) CV trends of the five electrodes from a single batch at 0.5 mg·mL^–1^ (Scan rate = 50 mV·s^–1^, Electrolyte: 5 mM RuHex in 0.1 M KCl). (d) Anodic peak current (*I*_pa_), (e) cathodic peak current (*I*_pc_), and (f) Δ*E*_p_ across three batches fabricated using 0.5 mg·mL^–1^ to demonstrate the reproducibility of *WAST* (mean ± standard error, *n* = 5 per batch).

As shown in Figure 3d-e, *“MXene-at-all-electrodes”* platform demonstrated good batch-to-batch consistency with *I*_pa_ = 87.9 ± 1.7 μA, *I*_pc_ = −81.3 ± 1.5 μA, and Δ*E*_p_= 255.9 ± 4.6 mV (mean ± SE, *n* = 15). Stable Δ*E*_p_ values (Figure 3f) combined with one-way analysis of variance (ANOVA) across three independent batches confirmed no statistically significant batch-to-batch differences (*p* > 0.05),^57^ thus indicating the reproducibility of the *WAST* approach for monolithic MXene platforms.

While this configuration serves as a material-centric platform to understand electrode architecture effects in a fully MXene-active environment, its electrochemical performance reveals inherent limitations. The observed capacitive dominance, Δ*E*_p_ broadening, and interelectrode polarization are the fundamental constraints of using MXene as a reference electrode. These findings compellingly demonstrate that while a monolithic platform is conceptually elegant, practical electrochemical sensing requires a stable potential reference and dedicated charge injection provided by conventional electrode materials, hence motivating the development of the hybrid architecture presented in the following section.

### Configuration B (MXene as the Working Electrode)

To further investigate the effects of electrode architecture and material loading on redox behavior, electrodes with a more standardized configuration (configuration B), with MXene serving exclusively as the working electrode, were fabricated. For this, different volumes (*1, 2, and 4 mL*) of MXene inks were filtered at a fixed low concentration (0.05 mg·mL^–1^). A lower concentration facilitated rapid filtration and improved the pattern quality by preventing MXene adsorption onto the waxed regions. CV trends in Figure 4a demonstrate differences in redox features (i.e., different mass-transport properties).^58^ Electrodes from the *1 mL* volume exhibited sluggish electron transfer due to inhomogeneous flake deposition with an extremely thin layer.^47^ Additionally, obtaining a uniform film formation at such a lower volume is challenging due to the hydrophobic nature of the wax membrane and the limited filtrate volume. Nonetheless, meticulous filtration can yield ultrathin (transparent) conductive electrodes. Whereas the electrode produced at a higher volume (4 mL) reveals enhanced redox kinetics and well-defined peaks, albeit with a modest increase in capacitive background (Figure 4b). In contrast, measurements performed in the absence of RuHex display no Faradaic features, confirming that the observed redox activity originates from the redox probe rather than the electrode itself. While increasing the volume leads to a thicker MXene film, it still allows efficient ion diffusion. As long as the film is not excessively thick, mass transport limitations do not dominate, and the system maintains efficient kinetics.

**Figure 4:**
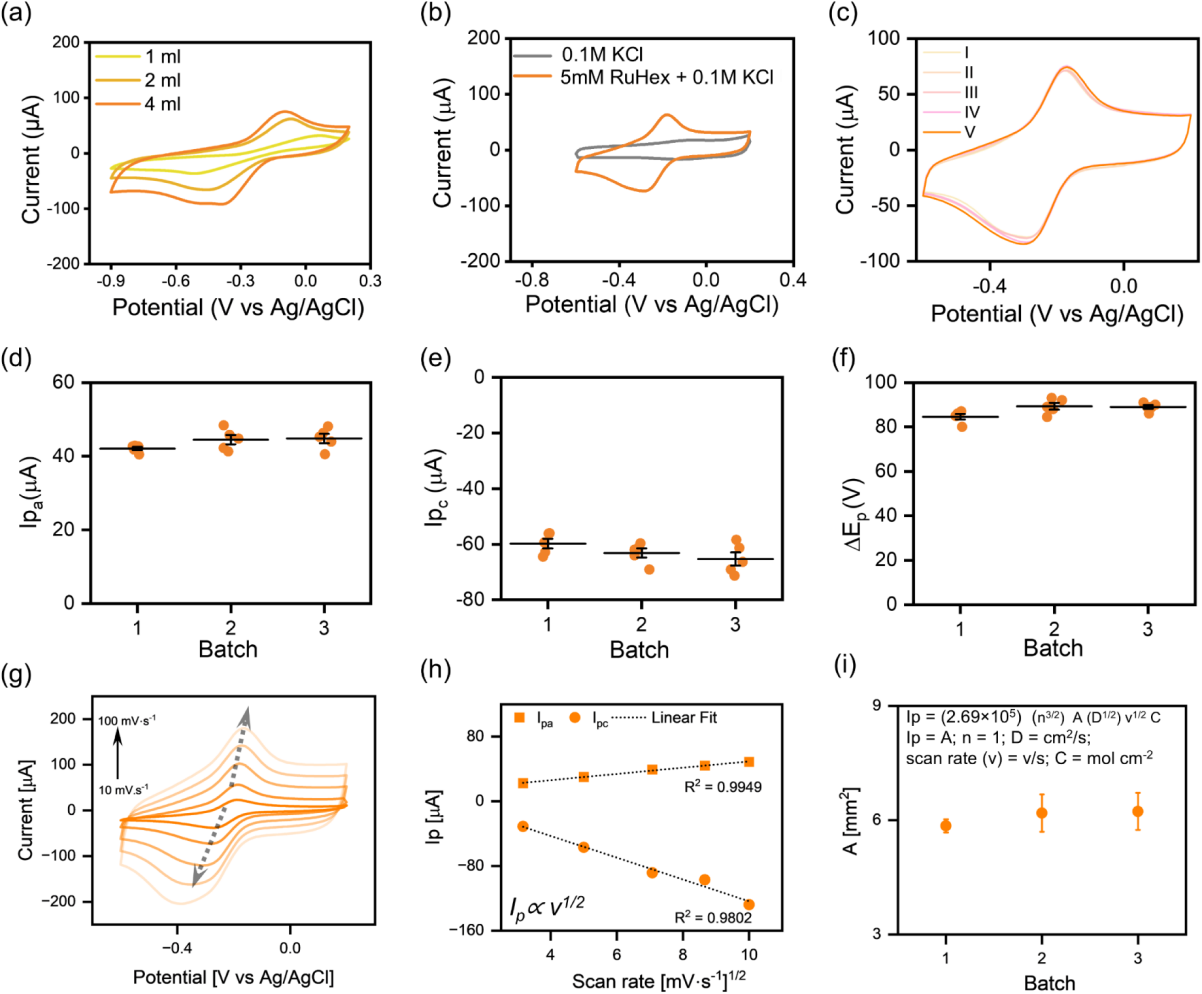
CV trends of (a) electrodes fabricated at 1, 2, and 4 mL of MXene suspensions (Scan rate = 50 mV·s^–1^, Electrolyte: 5 mM RuHex in 0.1 M KCl). (b) CV response of stamped electrodes with and without the RuHex redox probe (Scan rate = 50 mV·s^–1^). (c) Five electrodes from a single batch showing consistent redox behavior (Scan rate = 50 mV·s^–1^, Electrolyte: 5 mM RuHex in 0.1 M KCl). (d–f) Anodic peak current (*I*_pa_), cathodic peak current (*I*_pc_), and peak-to-peak separation (Δ*E*_p_) across different batches, confirming the reproducibility (mean ± standard error, *n* = 5 per batch). (g) CV trends of an electrode at different scan rates of 10, 25, 50, 75, and 100 mV·s^–1^. (h) The linear dependence of peak currents (*I*_pa_ and *I*_pc_) on the square root of scan rates (ν^1/2^) follows the Randles–Ševčík relationship. (i) Electrochemically active surface area of electrodes across batches (mean ± 95% confidence interval).

Also, by effectively mitigating the instability and possible interelectrode polarization effect, configuration B establishes clear structure–function relationships. This underscores a critical trade-off between thickness and performance (the capacitive–faradaic balance) and highlights a key design principle for MXene-based sensors. The stamped electrodes demonstrated excellent reproducibility with minimal variations among the devices produced in a single batch (Figure 4c). The hybrid electrode design (Configuration B) exhibited exceptional reproducibility with remarkably consistent peak currents, with *I*_pa_ = 43.8 ± 0.6 μA and *I*_pc_ = −62.8 ± 1.2 μA (Figure 4c-d) and substantially lower peak separation (i.e., Δ*E*_p_ = 87.6 ± 0.9 mV) as shown in Figure 4f. The clustered Δ*E*_p_ values across the batches underscore the stability advantage conferred by the integrated Ag/AgCl pseudo-reference electrode. One-way ANOVA further confirmed the reliability of the fabrication process, revealing no significant batch-to-batch variation (*p* > 0.05)^57^ among key parameters, e.g., peak currents.

The electrochemical kinetics of the MXene-stamped electrodes were further examined by varying the scan rate from 10 to 100 mV·s^–1^ (Figure 4g). Plotting the peak currents against ν^1/2^ showed a linear dependence of *I*_pa_ following the Randles–Ševčík equation, compared to a sublinear *I*_pc_ response (Figure 4h).^59^ Quantitatively, the ratio of diffusion coefficients (*D*_ox_/*D*_red_ ≈ 0.7) supports the different electron transfer of the oxidized and reduced species of the [Ru­(NH_3_)_6_]^3+/2+^ probe.^60^ Also, a *b*-value of ∼0.34 across the measured scan rates (10–100 mV·s^–1^), obtained by power-law analysis (*i* = *a*ν^*b*^, S4), indicate a deviation from diffusion-controlled behavior (*b* = 0.5).^61^ This suggests kinetic limitations within the electrode (e.g., hindered in-film ion accessibility, resistive and ohmic distortions, or coupled interfacial kinetics). To this end, electrochemical impedance spectroscopy (EIS) and/or multiple-step chronoamperometry (MUSCA)^62^ would provide complementary insights; however, such analyses are beyond the present focus on fabrication/configurability and will be addressed in follow-up work. This also implies an operational scan-rate limit for such devices; therefore, lower scan rates (<50 mV·s^–1^) are recommended, which provided a well-defined, quasi-reversible response (Δ*E*_p_ ≈ 87 mV). Nonetheless, voltammetry effectively assesses the overall electrochemical behavior of the fabricated electrodes and their fabrication-related aspects.

Similarly, the electrochemically active surface area (ECSA: 4.8 ± 0.3 mm^2^ ; *n* = 15) determined from Randles–Ševčík was uniform across the different batches (Figure 4i).^63^ The measured area is larger than the geometric surface area (GSA: 3.00 mm^2^) defined during device design, yielding a roughness factor (RF = ECSA/GSA) of 1.6.^64^ This enhancement (or roughness factor) can be attributed to the microstructural roughness/texture induced by wrinkles and probable interflake porosity among the stacked MXene sheets. Consistent ECSA combined with the previously observed uniform electrochemical performance further underscores the functional reliability of the *WAST* approach to fabricate viable electrochemical transducers.

#### Intra- and Interplatform Benchmarking

The intraplatform benchmarking of our two configurations revealed a crucial structure–function correlation. The monolithic, MXene-at-all electrode configuration (Configuration A) suggests a single-material device; however, its electrochemical performance is limited by the inherently unstable nature of the MXene reference electrode, which resulted in an inconsistent potential field and a nonuniform driving force for electron transport. In comparison, Configuration B notably resolves this limitation by isolating fundamental electrochemical operations. By combining the electrochemical activity of the MXene working electrode with a stable Ag/AgCl pRE and carbon CE for facile charge injection, Configuration B shows better reproducibility with much less variability across Δ*E*_p_ (3.9% RSD vs 7.0% in Configuration A). This structure–function insight confirms that the effective electrochemical performance is not just material-centric but a rationally designed system with specialized parts for distinct functions. This also underscores the reliability of our wax-assisted stamp-transfer fabrication method for lab-scale manufacturing of electrochemical platforms while enabling flexibility, configuration choice, and tool-light patterning.

Furthermore, the interplatform benchmarking rigorously evaluated the MXene-based electrode (Configuration B) against conventional carbon architecture, e.g., carbon screen-printed electrodes (CSPE). Similar to CSPE, the Configuration B exhibited a well-defined faradaic response (Figure 5a). Higher peak currents (*I*_pa_ and *I*_pc_) and smaller Δ*E*_p_ can be attributed to metal-like conductivity, electroactive surface, and stamp-induced morphological roughness. One may note that the enhanced capacitive response of MXenes contributes to the overall current profile. Nevertheless, the primary reason for this comparison lies in validating the functionality of the *WAST* method to produce custom-made electrodes capable of functioning equally well as a commercial system while sustaining efficient electron transfer.

**Figure 5:**
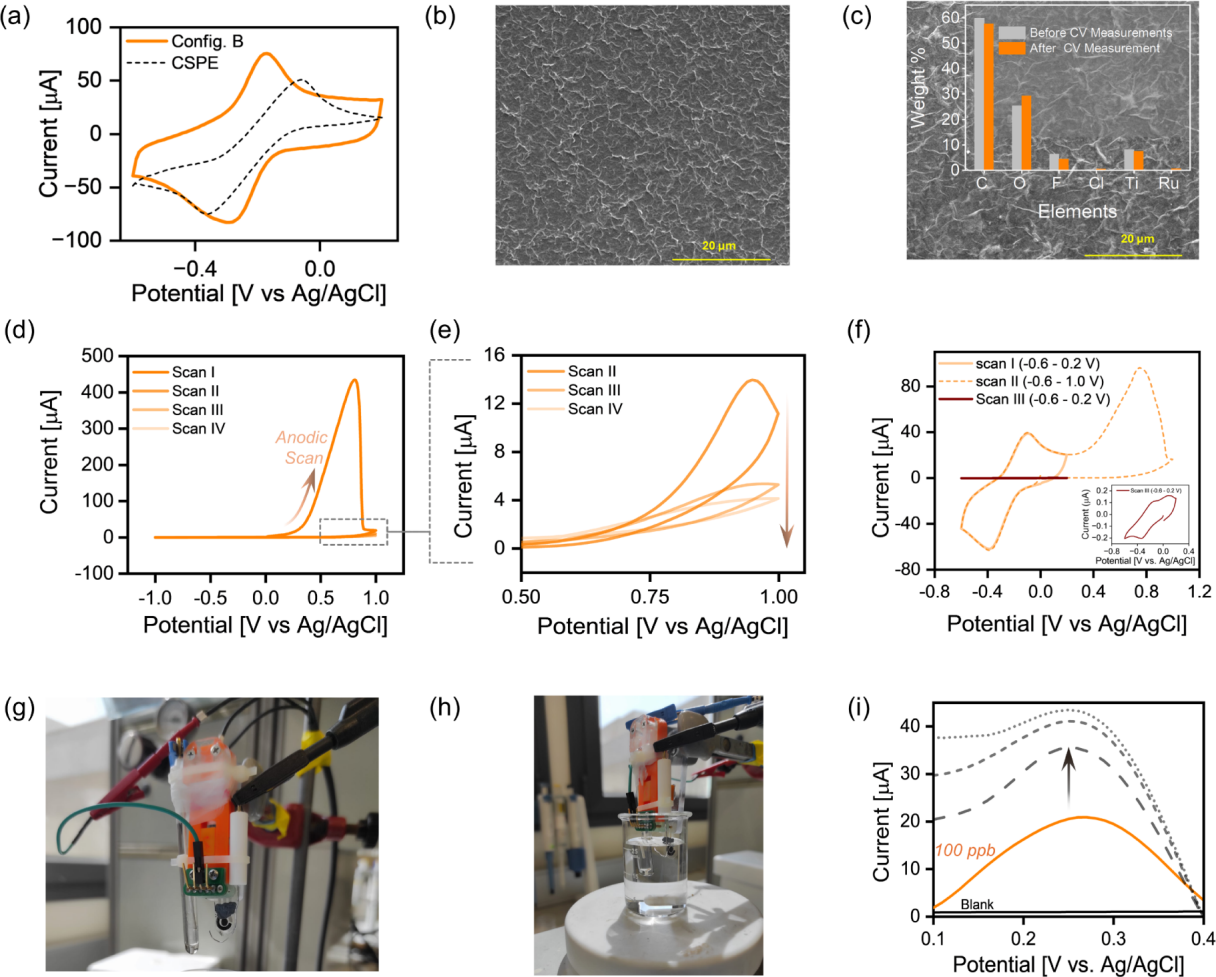
(a) Comparative cyclic voltammograms of Configuration B and carbon screen-printed electrodes (CSPE) at 50 mV·s^–1^ (Electrolyte: 5 mM RuHex in 0.1 M KCl). SEM images of MXene electrode (b) before and (c) after CV measurements showing intact morphology; inset shows EDXS composition. (d) Four consecutive voltammetric scans in the positive potential window. (e) Magnified view of scans II–IV showing a gradual decrease in the electrochemical signal after scan I (Electrolyte: 0.1 M KCl). (f) First three CV scans of MXene electrodes in 5 mM RuHex in 0.1 M KCl show irreversible redox behavior after scanning in the extreme positive potential window. (g,h) Digital photographs of a heavy metal ion sensing experiment, where the working electrode was paired with a platinum (Pt) counter electrode and Ag/AgCl pseudoreference electrode. (i) The square-wave voltammetry trends for Hg^2+^ concentrations show a gradual increase in the current with increasing concentration under a deposition time of 120 s, an E-step of 0.01 V, 5 Hz, and baseline correction.

#### Morphological Stability, Operating Window, and Preliminary Application

SEM images showed that the stamped MXene electrodes remained intact without voids (or detachment) before and after several voltammetric scans (Figure 5b-c). In addition, the EDX elemental mapping in the inset shows minimal ruthenium residues, as expected from the positively charged electrolyte adsorption over the negatively charged MXene surface.^55^ This underscores the critical importance of the electrode architecture, as the implementation of a carbon-based CE (instead of MXene CE) mitigates detrimental polarization effects (observed in Configuration A). Therefore, the improved design directly yields both superior mechanical integrity and more robust electrochemical operation.

The determination of a feasible potential window is paramount to assessing the electrochemical stability of stamped electrodes. A substantial anodic peak (in 0.1 M KCl) at a potential of 0.8 V vs Ag/AgCl pRE, observed during the first anodic scan (Figure 5d), indicates the oxidation of the electrode material itself, and the absence of a reduction peak suggests its irreversible nature. Presumably, the formation of TiO_2_ on the MXene surface led to a gradual decrease in the oxidation current in subsequent scans (Figure 5e). Following the onset (approximately +0.3 V) of material oxidation, we limit the scanning window up to +0.2 V so as not to deteriorate the electrode. To observe the progression of this effect in the presence of redox couple, CV curves recorded in stable potential windows (Scan-I; −0.6 to +0.2 V) show the expected peaks of Ru^3+^/Ru^2+^ couple (Figure 5f). However, expanding the window to a more positive range (Scan-II; −0.6 to +1.0 V) reveals an additional anodic peak at +0.8 V. The subsequent rescan (Scan-III) in the stable potential window did not yield a CV trend similar to Scan-I (Figure 5f inset).^52^ This indicates that the electrochemically driven oxidation process induces structural changes and impairs the redox reproducibility of the electrodes. This thereby establishes an operational limitation and prevent higher positive windows for pristine MXene electrodes.

To assess the functionality of the fabricated electrodes for future sensing devices, we evaluated their performance for heavy-metal ion sensing via square-wave anodic stripping voltammetry (SWASV). For this, MXene working electrodes were integrated into a conventional three-electrode system employing a stable, true Ag/AgCl reference electrode and a platinum wire counter electrode (Figure 5g,h) and tested in a 0.1 M KCl solution. A well-resolved oxidation peak at ∼0.27 V in the first analysis cycle is presented here (Figure 5i), which corroborates earlier reports^65^ and is presented here as a *qualitative proof-of-concept* for Hg^2+^ detection. While further investigations of the detection profile and cleaning protocols to mitigate any memory effects are required, this initial effort highlights the potential of *WAST*-fabricated electrodes for sensing applications. Future studies could focus on interface engineering, such as exploiting the self-reduction of MXenes^66^ or applying other solvent-free, one-step strategies^67^ for a wide range of applications. This study therefore lays the groundwork for broadening the functionality of MXenes for electrochemical sensing.

## Conclusions

*WAST* is an additive-free approach to pattern and transfer MXene films onto flexible substrates. Comprehensive structural and chemical investigations (XRD, SEM, TEM/STEM, XPS, and EDS) accompanied by colloidal characterization (DLS, zeta potential, and UV–vis) reveal high-quality Ti_3_C_2_T_*x*_ MXene with intact material properties. We identified a fundamental trade-off: while higher mass loadings increase the active material, they introduce significant capacitive background and ion transport limitations (*ΔE*_p_ = 255.9 ± 4.6 mV for MXene-at-all electrodes’ configurations). Conversely, a rationally designed hybrid structure significantly improved the electrochemical reversibility (Δ*E*_p_ = 87.6 ± 0.9 mV) with enhanced redox kinetics and superior performance compared to standard counterparts (e.g., CSPE). Our analysis revealed that configuration-B exhibits quasi-reversible behavior at lower scan rates, compared to kinetic limitations and diffusion barriers at elevated rates, which EIS or MUSCA would resolve and provide complementary deconvolution of true Faradaic signals and interfacial analysis. Also, nanoarchitectural engineering via intercalation strategies, composite formation, or alternative porous substrates can further prove beneficial. Nevertheless, the demonstrated *WAST* patterning strategy and the mechanistic insights from material-centric structure–property relationships provide a foundation for rational engineering of MXene-based interfaces for sensitive sensing platforms and related electrochemical technologies.

## Experimental Section

### Materials and Equipment

Ti_3_AlC_2_ MAX precursor was purchased from Carbon-Ukraine (particle size ≤ 40 μm). Hydrofluoric acid (HF, 48–51%) was obtained from ACROS Organics. Hydrochloric acid (HCl, ≥37%, ACS Reagent) and lithium chloride (LiCl, 99%) were purchased from Sigma-Aldrich. All of the reagents were used without further purification. Polyethylene terephthalate (PET) sheets were used for electrode fabrication. Screen printing of the silver-ink electrical contacts, Ag/AgCl pseudoreference electrodes, and carbon counter electrodes was performed using a screen printer, whereas the working electrodes were transferred using a hydraulic press. A poly­(vinylidene difluoride) (PVDF) hydrophobic membrane (0.1 μm pore size, 47 mm diameter) from Millipore was used for wax printing and subsequent vacuum filtration. Wax printing was performed using a Xerox ColorQube 8580. Vacuum filtration was performed using a 1 L vacuum-filtering flask equipped with a 300 mL glass filter holder and a 47 mm filtration membrane from Millipore, attached to a vacuum pump.

### MAX Etching and Delamination

First, 1 g of Ti_3_AlC_2_ (MAX Phase) was slowly added to an etching solution of DI-H_2_O + HCl + HF (9 mL + 18 mL + 3 mL) and stirred at 300–400 rpm for 24 h at 35 °C. The etched product was then washed with Milli-Q water by centrifugation (3500 rpm, 5 min) until the pH of the supernatant reached approximately 6–7 and subsequently collected by vacuum filtration. For delamination, 1 g of LiCl powder was dissolved in 50 mL of Milli-Q water with minimal stirring. The etched MXene sediment was then added and stirred at 300 rpm for 24 h at 35 °C under a continuous argon flow. The mixture was washed 2–3 times with Milli-Q water at 3500 rpm for 5 min. When the supernatant turned dark, the centrifugation time was increased to 1 h to obtain a clear supernatant. The sediment at the bottom, which swelled significantly, was referred to as MXene clay. The clay was further dispersed in Milli-Q water and mixed with a glass rod, followed by gentle shaking (for a few minutes) until a homogeneous suspension was obtained. To separate the delaminated single-layer MXene (d-MXene), the mixture containing unreacted MAX and multilayer (ML) and single-layer MXene was centrifuged at 3500 rpm for 20 min, yielding a viscous, dark black supernatant that was carefully collected. To ensure further purity, the collected supernatant was centrifuged at 3500 rpm for 20 min, and the supernatant containing single layers of MXenes was collected. Before storage, the vials containing MXene were purged with argon gas and stored at 4 °C until further use. The remaining sediment, which consisted of unetched MAX and ML-MXene, was discarded.

### Electrode Fabrication

As shown in S5, two electrode configurations (named Configuration A and Configuration B) were designed using AutoCAD 2018 (Autodesk, USA) and printed using the wax printer in high-resolution mode. Before filtration, a tubular spirit level was used to ensure uniform collection of active material in non-waxed areas. Filtration begins with the formation of an initial layer of MXene flakes in the wax-free regions. As filtration proceeds, more flakes are accumulated; consequently, the filtration rate decreases. This implies that the concentration and filtrate volume are important to obtain a stampable film with fair pattern resolution. For example, if high concentrations or volumes are used, it causes rapid accumulation, uneven coverage, and compromised resolution of the patterns, whereas the low volume or concentration may produce thin coatings or uncovered areas. This means that the *WAST* approach requires appropriate balance of filtration’s concentration and volume. After filtration, the membranes containing captured MXene were cut to the desired size (one electrode each time). The back of each membrane was wetted with deionized water and placed with the MXene side up on a bi-adhesive tape affixed to an A4 sheet strip. The connectors were cleaned using an O-plasma, aligned over the membrane, and sandwiched with another strip of A4 paper. The transfer was then performed using a hydraulic press by applying a force of 10 tons for 2 min to secure the MXene electrode onto the host substrate. The adhesion of MXene was robust, as the final electrodes withstood standard procedures (rinsing with deionized water and drying with a nitrogen stream) without any signs of material loss. Digital photographs at each step of the fabrication are presented in part S6. Note that each membrane contained five electrodes.

### Characterization Details

X-ray diffraction (XRD) data were collected using a PANalytical X’Pert Pro multipurpose diffractometer (MPD) in Bragg–Brentano geometry with Cu Kα radiation (λ = 1.54 Å). Scanning electron microscopy (SEM) analysis was performed using a Thermo Fisher Scientific XHRSEM Magellan 400 L microscope. Energy-dispersive X-ray spectroscopy (EDX) mapping was conducted by using a Quanta 650 FEG system following the SEM imaging. A diluted suspension of d-Ti_3_C_2_T_*x*_ (<0.05 mg/mL) was drop-casted onto an anodic alumina (AAO) substrate before acquiring top-view SEM images. Dynamic Light Scattering (DLS) and zeta potential measurements were performed using a Zetasizer Nano ZS (Malvern Instruments) with disposable cuvettes, employing automatic focus calibration and analysis. Three measurements were performed to obtain the average DLS values. The surface elemental composition of MXenes was characterized by X-ray photoelectron spectroscopy (XPS) using a near-ambient pressure (NAP-XPS) system equipped with a PHOIBOS 150 1D-DLD analyzer. The spectrum was analyzed using CasaXPS software. Resistance measurements were performed using a Keithley DMM 6500 digital multimeter (S7). For electrochemical measurements, a solution of 5 mM Hexaammineruthenium­(III) chloride ([Ru (NH_3_)_6_]­Cl_3_) in 0.1 M potassium chloride (KCl) was used as a redox-active. The following equations follow the oxidation and reduction of RuHex probe and Δ*E*_p_ calculations:^59^

$$\mathrm{Oxidation:}\;{\left[{\mathrm{Ru}\left({\mathrm{NH}}_{3}\right)}_{6}\right]}^{2+}\to {\left[{\mathrm{Ru}\left({\mathrm{NH}}_{3}\right)}_{6}\right]}^{3+}+e^{-}$$

$$\mathrm{Reduction:}\;{\left[{\mathrm{Ru}\left({\mathrm{NH}}_{3}\right)}_{6}\right]}^{3+}+e^{-}\to {\left[{\mathrm{Ru}\left({\mathrm{NH}}_{3}\right)}_{6}\right]}^{2+}$$

$$\mathrm{Peak}‐\mathrm{to}‐\mathrm{Peak}\;\mathrm{Potential}\;\mathrm{Difference}\;{\Delta E}_{p}=E_{\mathrm{pa}}-E_{\mathrm{pc}}$$

Briefly, the electrodes were connected to a potentiostat via a commercial edge connector module, and 100 μL of the electrolyte solution was drop-cast (see S8). The PalmSens 4 potentiostat was used in conjunction with the PSTrace software (version 5.9). Electrochemical data are presented as the mean ± standard error of the mean (SEM), which is used to represent measurement uncertainty. Statistical analysis was performed using OriginPro, and scatter plots were produced to visualize the distribution of each data point along with the mean ± SEM. Reproducibility between different production batches was assessed using one-way ANOVA with Tukey’s post hoc test for multiple comparisons. In this study, three independent production batches were analyzed for each configuration, with *n* = 5 electrodes per batch, and statistical significance was defined as *p* < 0.05.

## Supplementary Material



## Notes

UV–vis spectroscopy and dynamic light scattering (S1); Energy-dispersive X-ray spectroscopy (S2); X-ray photoelectron spectroscopy survey spectra (S3); Power law analysis (S4); Electrode design configurations (S5); Digital photographs at every step of fabrication (S6); Electrical characterization of stamped electrodes (S7); Experimental setup for electrochemical characterizations (S8) (PDF)
