# Supplementary material for: Filter, Flip, and Fabricate: A Wax-Assisted Stamp-Transfer Approach for Flexible Ti3C2T x MXene Electrochemical Transducers
Source: ACS Appl Mater Interfaces. 2026 Jan 15;18(3):5791–802. doi: 10.1021/acsami.5c24165 (PMC12862758; doi:10.1021/acsami.5c24165)
Supplement: Supplementary file 1 [file am5c24165_si_001.pdf]

Supporting Information

Filter, Flip, and Fabricate: Wax-Assisted Stamp Transfer Approach for $Ti_3C_2T_x$ MXene Flexible Electrochemical Transducers

*Zaheer Ud Din Babar [a,b], Andy Bruno [a,c], Gabriel Maroli [a], Syedah Afsheen Zahra [d], Bartolomeo Della Ventura [e], Raffaele Velotta [e], Vincenzo Iannotti [f *], Ruslán Alvarez-Diduk [a *], Arben Merkoçi [a,g *]*

a Catalan Institute of Nanoscience and Nanotechnology (ICN2), CSIC and BIST, Campus UAB, 08193 Bellaterra, Barcelona, Spain.

b Scuola Superiore Meridionale (SSM), University of Naples Federico II, Largo S. Marcellino, 10, 80138, Italy. Department of Physics "E. Pancini", University of Naples Federico II, Via Cintia 26, 80126, Naples, Italy

c Autonomous University of Barcelona (UAB), Bellaterra, Barcelona, Spain

d Laboratory for Functional Polymers, EMPA Swiss Federal Laboratories for Materials Science and Technology, Überlandstrasse 129, 8600, Dübendorf, Switzerland.

e Department of Physics "E. Pancini", University of Naples Federico II, Via Cintia 26, 80126, Naples, Italy

f Department of Physics "E. Pancini", University of Naples Federico II, Via Cintia 26, 80126, Naples, Italy ; CNR-SPIN c/o Department of Physics "E. Pancini" Piazzale V. Tecchio 80, 80125,Naples, Italy.

g ICREA Catalan Institution for Research and Advanced Studies (ICREA), Barcelona, Spain

** Email: viannotti@unina.it, ruslan.alvarez@icn2.cat , arben.merkoci@icn2.cat*

S1. UV-Vis Spectroscopy and Dynamic Light Scattering

The surface of $Ti_3C_2T_x$ is typically terminated with functional groups such as -OH, -F, and =O, which influence its electronic structure, and consequently, its optical absorption properties and surface charge. The interactions between these surface terminations and the MXene layers can lead to variations in the absorption characteristics, resulting in distinctive UV–vis spectra absorption peak around 780nm. [1] Meanwhile, it is important to note that the particle size determined by dynamic light scattering (DLS) represents the hydrodynamic diameter of the flakes, which may not exactly correspond to the actual flake size. Nevertheless, DLS provides a reliable estimate of flake dimensions, as widely reported in the literature. [2]

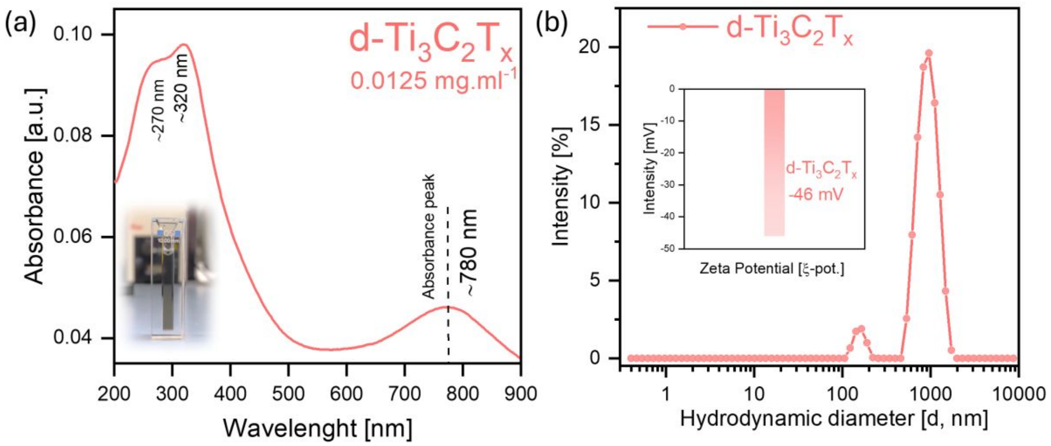

Figure S1. (a) UV-visible absorption spectrum of delaminated $Ti_3C_2T_x$ MXene. **(b)** Dynamic light scattering (DLS) size distribution of MXene colloidal suspensions, with the inset showing the corresponding zeta potential (ζ).

S2. Energy-Dispersive X-ray Spectroscopy

The Energy-Dispersive X-ray Spectroscopy (EDXS) spectra demonstrates the complete removal of the Al peak from the MAX phases upon conversion to delaminated MXene, confirming the production of high-quality MXene with no residual aluminum [3]. The relatively high oxygen content is attributed to inevitable oxygen terminations introduced during the acid etching process. It is important to note that a small amount of Al (as shown in the inset) is still present in multilayer $Ti_3C_2T_x$ (ml-$Ti_3C_2T_x$), typically due to AlF_x and unreacted MAX phases. However, this residual aluminum disappears after delamination process, where unreacted MAX and AlF_x are effectively removed through extensive washing and separation steps.[4]

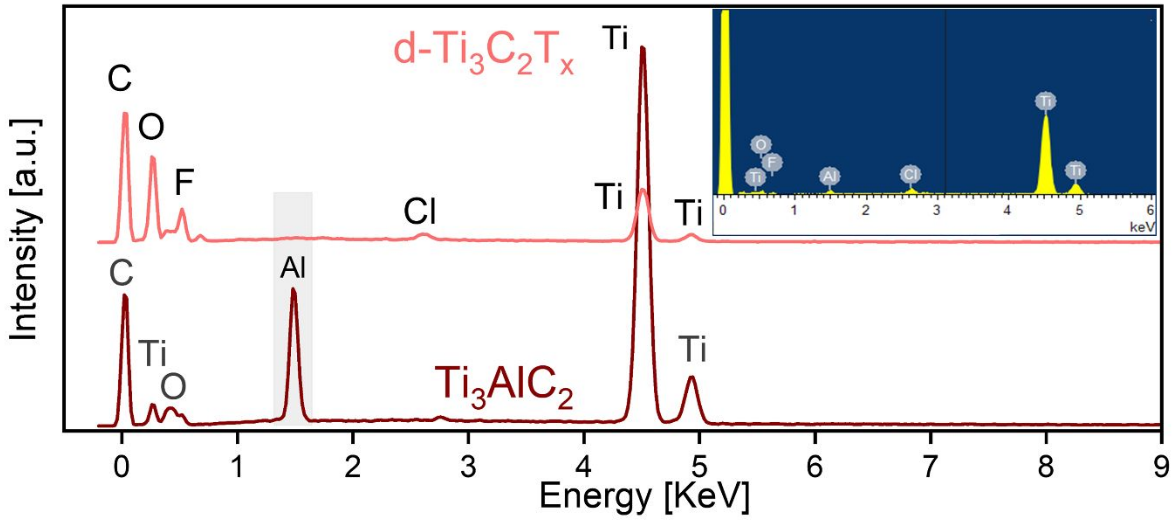

Figure S2. EXD elemental spectra of Ti_3AlC_2 MAX and delaminated $Ti_3C_2T_x$ MXene while the elemental composition of ml-$Ti_3C_2T_x$ is shown in the inset.

S3. X-ray Photoelectron Spectroscopy Survey Spectra

X-ray Photoelectron Spectroscopy (XPS) survey spectra were acquired to investigate the elemental composition and surface chemistry of the delaminated Ti_3C_2Tx MXene. The wide-scan spectra shown in Fig S-3 confirmed the presence of titanium (Ti), carbon (C), oxygen (O), fluorine (F), and Chlorine (Cl) without any aluminum (Al) content, indicating the successful removal of Al from the parent MAX phase [5]. The peaks corresponding to Ti 2p, C 1s, O 1s, and F 1s were clearly identified and were consistent with the expected surface terminations (=O, -OH, -F) commonly found in acid-etched MXenes. [6]

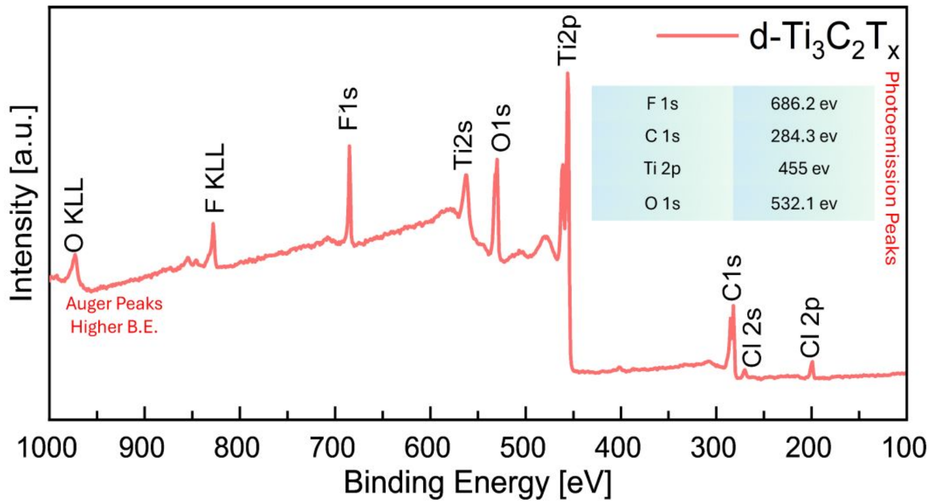

Figure S3. XPS survey spectra of d-$Ti_3C_2T_x$ MXene.

S4. Power Law Analysis:

In cyclic voltammetry, the power-law relationship is expressed as;

$$i = i_{\text{diff.}} + i_{\text{cap.}} = av^b$$

and signify that the total current (i) is the sum of the capacitive and faradaic contributions. where v is the scan rate, and a and b are the adjustable parameters. The parameter b is determined from the slope of log(i) versus log (v) as follows:

$$\log (i) = \log (a) + \mathrm{b} \log (v)$$

A b-value of 0.5 indicates that the current is limited by ion diffusion from the bulk electrolyte to the electrode surface (a semi-infinite diffusion-controlled faradaic process). A b-value of 1.0 indicates fast surface-confined reactions, such as electric double-layer charging (EDLC) or pseudocapacitive surface redox reactions. Experimentally, a b-value between 0.5 and 1 signifies a mixed process.[7] A value below 0.5 indicates a deviation from ideal semi-infinite diffusion, which is often associated with a finite diffusion confined to electrode architecture.[8]

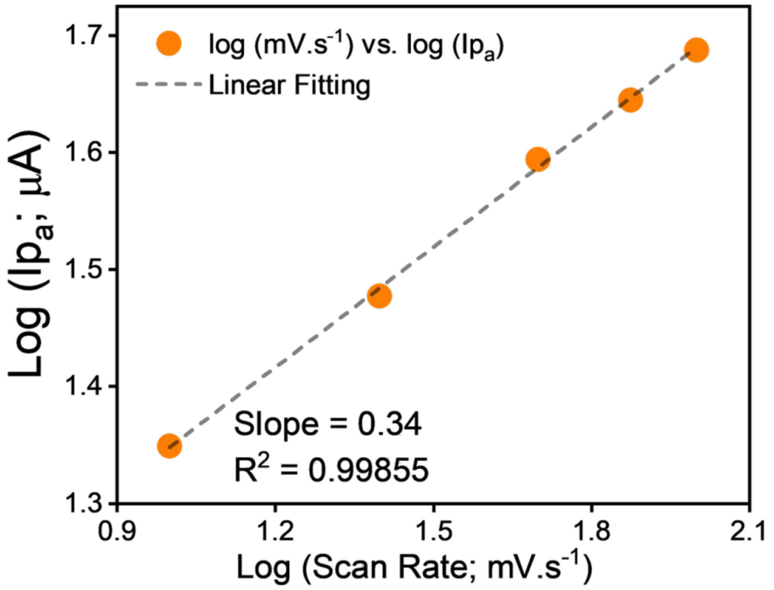

Figure S4: Power law analysis of Stamped MXene working electrode

S5. Electrode Design Configurations

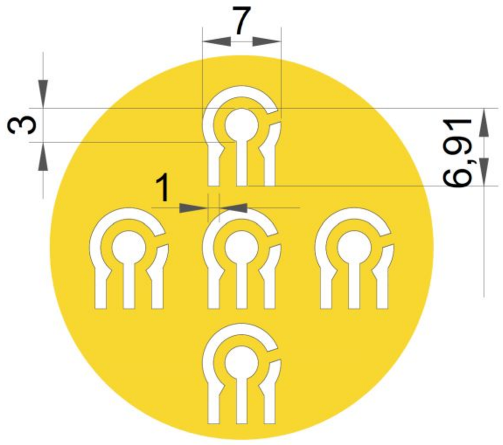

Figure S5. Configuration A of electrode designs with relevant dimensions (all units are in mm). Please note the rest of the details in Configuration B that contains only the working electrode are the same.

S6. Digital photographs at every step of fabrication

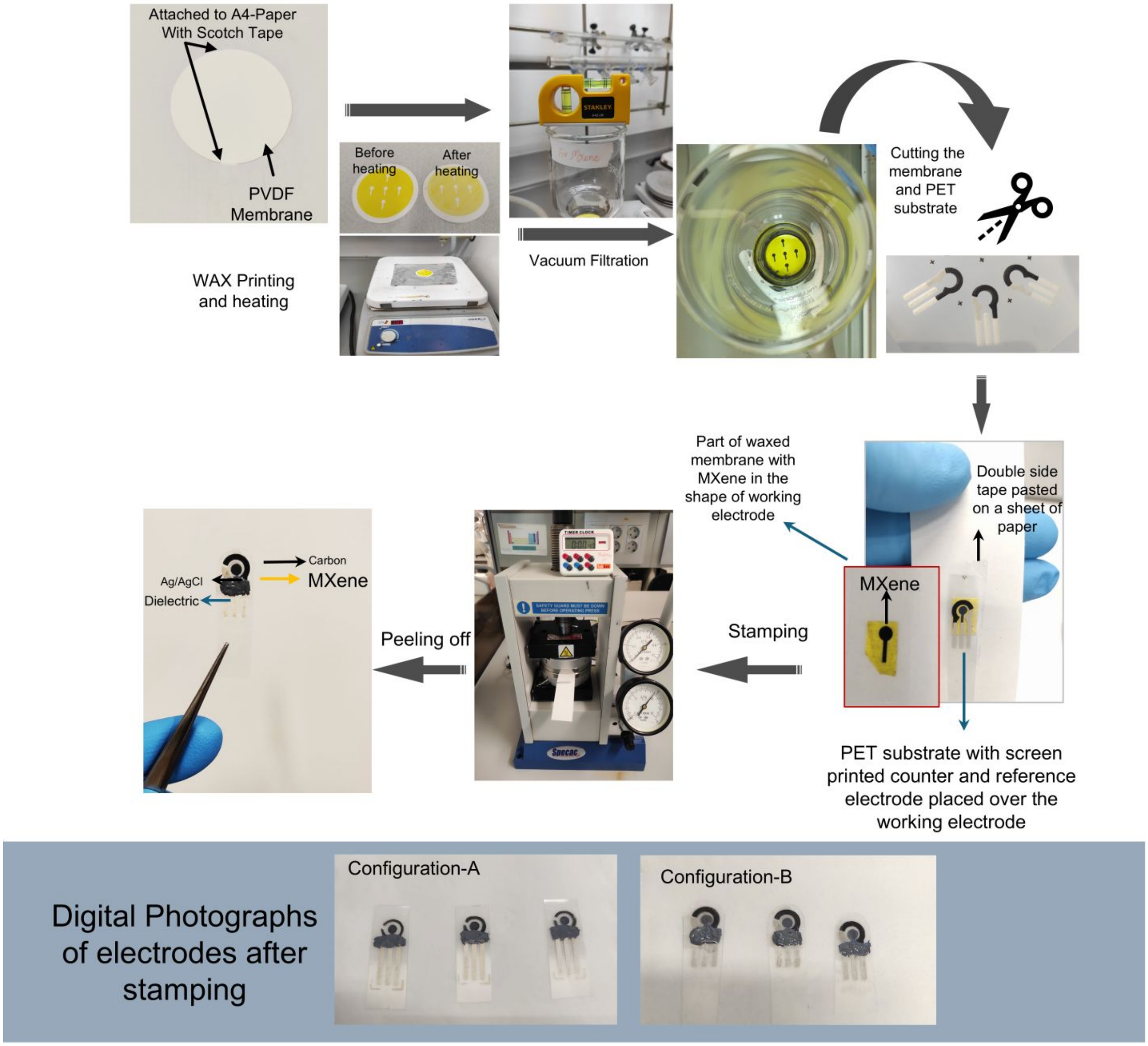

Figure S6. Digital photographs illustrating each step of the electrode fabrication

S7. Electrical Characterization of Stamped Electrodes

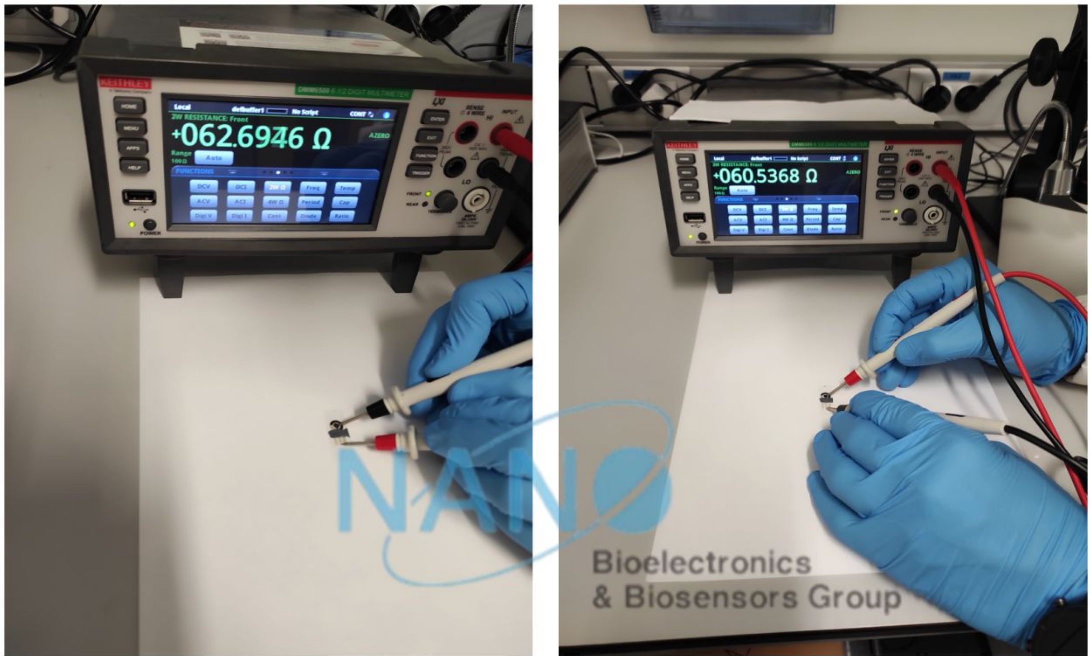

Figure S7. Contact resistance of two stamped electrodes after blocking and overnight drying.

S8. Experimental Setup for Electrochemical Characterizations

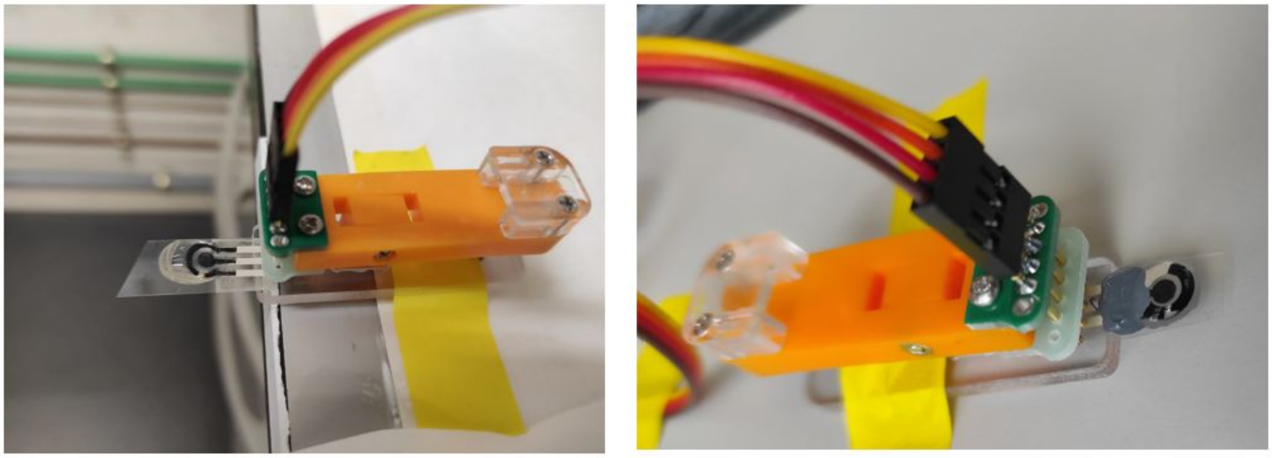

Figure S8: Digital Photographs of electrodes mounted on commercial edge connector.

References:

1. Sunderiya, S.; Suragtkhuu, S.; Purevdorj, S.; Ochirkhuyag, T.; Bat-Erdene, M.; Myagmarsereejid, P.; Slattery, A. D.; Bati, A. S. R.; Shapter, J. G.; Odkhuu, D.; Davaasambuu, S.; Batmunkh, M., Understanding the oxidation chemistry of Ti3C2Tx (MXene) sheets and their catalytic performances. *Journal of Energy Chemistry* **2024,** *88*, 437-445.
2. Maleski, K.; Ren, C. E.; Zhao, M.-Q.; Anasori, B.; Gogotsi, Y., Size-Dependent Physical and Electrochemical Properties of Two-Dimensional MXene Flakes. *ACS Applied Materials & Interfaces* **2018,** *10* (29), 24491-24498.
3. Tsyganov, A.; Shindrov, A.; Vikulova, M.; Zheleznov, D.; Gorokhovsky, A.; Gorshkov, N., Effect of LiCl Electrolyte Concentration on Energy Storage of Supercapacitor with Multilayered Ti3C2Tx MXene Electrodes Synthesized by Hydrothermal Etching. Processes **2023,** *11* (9), 2528.
4. Renkler, N. Z.; Babar, Z. U. D.; Barra, M.; Cruz-Maya, I.; De Santis, R.; Girolamo, R. d.; Marelli, M.; Ferretti, A. M.; Zaheer, A.; Iannotti, V.; Guarino, V., Optimizing Electrospun PVA Fibers with MXene Integration for Biomedical Applications. *Macromol. Mater. Eng.* **2025,** *310* (7), 2400433.
5. Guo, Y.; Zhou, X.; Wang, D.; Xu, X.; Xu, Q., Nanomechanical Properties of Ti3C2 Mxene. *Langmuir* **2019,** *35* (45), 14481-14485.
6. Natu, V.; Benchakar, M.; Canaff, C.; Habrioux, A.; Célérier, S.; Barsoum, M. W., A critical analysis of the X-ray photoelectron spectra of Ti3C2Tz MXenes. *Matter* **2021,** *4* (4), 1224-1251.
7. Liu, J.; Wang, J.; Xu, C.; Jiang, H.; Li, C.; Zhang, L.; Lin, J.; Shen, Z. X., Advanced Energy Storage Devices: Basic Principles, Analytical Methods, and Rational Materials Design. Adv. Sci. **2018,** *5* (1), 1700322.
8. Chagnot, M.; Abello, S.; Wang, R.; Dawlaty, J.; Rodríguez-López, J.; Zhang, C.; Augustyn, V., Influence of Finite Diffusion on Cation Insertion-Coupled Electron Transfer Kinetics in Thin Film Electrodes. *Journal of The Electrochemical Society* **2024,** *171* (1), 010527.
